# Laterally Extended Endopelvic Resection Versus Chemo or Targeted Therapy Alone for Pelvic Sidewall Recurrence of Cervical Cancer

**DOI:** 10.3389/fonc.2021.683441

**Published:** 2021-05-25

**Authors:** Soo Jin Park, Jaehee Mun, Seungmee Lee, Yanlin Luo, Hyun Hoon Chung, Jae-Weon Kim, Noh Hyun Park, Yong Sang Song, Hee Seung Kim

**Affiliations:** ^1^ Department of Obstetrics and Gynecology, Seoul National University College of Medicine, Seoul, South Korea; ^2^ Department of Obstetrics and Gynecology, Keimyung University School of Medicine, Daegu, South Korea; ^3^ Department of Gynecologic Oncology, Affiliated Cancer Hospital of Zhengzhou University (Henan Cancer Hospital), Zhengzhou, China

**Keywords:** laterally extended endopelvic resection, pelvic sidewall recurrence, survival, pain, cervical cancer

## Abstract

**Background:**

Laterally extended endopelvic resection (LEER) has been introduced for treatment of pelvic sidewall recurrence of cervical cancer (PSRCC), which occurs in only 8% of patients with relapsed cervical cancer. LEER can only be performed by a proficient surgeon due to the high risk of surgical morbidity and mortality, but there is no evidence as to whether LEER is may be more effective than chemo or targeted therapy alone for PSRCC. Thus, we aimed to compare the efficacy and safety between LEER and chemo or targeted therapy alone for treatment of PSRCC.

**Methods:**

We prospectively recruited patients with PSRCC who underwent LEER between December 2016 and December 2019. Moreover, we retrospectively collected data on patients with PSRCC who received chemo or targeted therapy alone between January 2000 and December 2019. We compared treatment-free interval (TFI), progression-free survival (PFS), treatment-free survival (TFS), overall survival (OS), tumor response, neurologic disturbance of the low extremities, and pelvic pain severity in the different patient groups.

**Results:**

Among 1295 patients with cervical cancer, we included 28 (2.2%) and 31 (2.4%) in the prospective and retrospective cohorts, respectively. When we subdivided all patients into two groups based on the median value of prior TFI (PTFI, 9.2 months), LEER improved TFI, PFS, TRS and OS compared to chemo or targeted therapy alone (median, 2.8 *vs.* 0.9; 7.4 *vs.* 4.1; 30.1 *vs.* 16.9 months; P ≤ 0.05) in patients with PTFI < 9.2 months despite no difference in survival in those with PTFI ≥ 9.2 months, suggesting that LEER may lead to better TFI, PFS, TRS and OS in patients with PTFI < 9.2 months (adjusted hazard ratios, 0.28, 0.27, 0.44 and 0.37; 95% confidence intervals, 0.12-0.68, 0.11-0.66, 0.18-0.83 and 0.15-0.88). Furthermore, LEER markedly reduced the number of morphine milligram equivalents necessary to reduce pelvic pain when compared with chemo or targeted therapy alone.

**Conclusion:**

Compared to chemo or targeted therapy alone, LEER improved survival in patients with PSRCC and PTFI < 9.2 months, and it was effective at controlling the pelvic pain associated with PSRCC.

**Trial Registration:**

ClinicalTrials.gov, identifier NCT02986568.

## Introduction

Pelvic exenteration can be attempted as a cure for central recurrence of cervical cancer, which is seen in 10.7% of patients with disease recurrence after radical treatment such as radiotherapy and radical hysterectomy. Vaginectomy provides another option for patients with isolated vaginal recurrence with acceptable postoperative complications and quality of life compared to radiotherapy or pelvic exenteration (1, 2). The five-year survival rate of such patients ranges from 30 to 60% (3). On the other hand, pelvic sidewall recurrence of cervical cancer (PSRCC) is relatively rare, occurring in 8.3% of patients with disease recurrence (4). However, tumors invading the pelvic sidewall structure are not easy to remove by pelvic exenteration, and residual tumors after pelvic exenteration are associated with poor prognosis (5, 6). Since salvage radiotherapy reportedly fails to treat loco-regional tumors in a previously irradiated field, palliative chemotherapy is mainly used to slow disease progression and control the pelvic pain caused by tumor invasion in the pelvic sidewall structure (3, 4).

Laterally extended endopelvic resection (LEER), an ultra-radical surgery that aims to remove pelvic sidewall tumors, has been used since 1999 in an effort to improve patient survival (7). Based on the ontogenetic compartment theory, LEER can provide tumor-free margins (R0) by resecting tumors that propagate through multi-compartmental borders between the pelvic floor and sidewall muscles and the internal iliac vessel system (8). However, LEER is a highly skilled surgery that can only be done by a proficient surgeon. It requires definite anatomical knowledge of pelvic sidewall structure due to the risk of massive bleeding during resection of tumors invading the major pelvic vessels, and adhesion and fibrosis in a previously debulked or irradiated pelvis can increase surgical morbidity and mortality (9, 10).

Despite these limitations, LEER reportedly produces a five-year survival rate of about 50%, and the number of studies on the feasibility of LEER for selected patients with PSRCC has gradually been increasing since 2015 (6, 9–14). However, the criteria for identification of patients for whom LEER may be beneficial remain ambiguous, and there is no evidence as to whether LEER may be more effective than palliative chemo or targeted therapy alone. This is an especially important question considering the high number of morbidities related to LEER. Thus, we performed a prospective cohort study to evaluate the efficacy and safety of LEER for patients with PSRCC and investigated the criteria for selection of patients who may benefit from LEER compared to chemo or targeted therapy alone.

## Materials and Methods

### Study Design

We prospectively collected data on patients with PSRCC who underwent LEER in Seoul National University Hospital between December 2016 and December 2019. The study protocol was registered on ClinicalTrials.gov (NCT02986568) before any patients. For the prospective cohort study, we consecutively recruited patients who were aged 20 years or older; had Eastern Cooperative Oncology Group (ECOG) performance status of 0 or 1; had recurrent or refractory cervical cancer; had unilateral PSRCC not involving the greater sciatic foramen with or without uncontrolled pelvic pain despite sufficient opioid usage; had PSRCC that might be cured or uncontrolled pelvic pain that might be relieved by LEER; signed the approved informed consent form; and had no other treatment options except for LEER. We excluded patients who were under 20 years of age; had ECOG performance status of 2 or more; had bilateral PSRCC; had a treatment option other than LEER; or refused to sign the approved informed consent form.

As historical controls, we retrospectively collected data on patients with PSRCC who received chemo or targeted therapy alone without LEER between January 2000 and December 2019. The inclusion and exclusion criteria for the retrospective group were the same as those for the prospective group except that informed consent was not necessary. For both cohorts, we collected data such as patient age; histologic type; size of pelvic sidewall tumors on imaging studies; disease extent according to TNM stage on radiologic imaging studies (15); topographic location and direction of pelvic sidewall tumors; types of prior treatment; tumor response to prior treatment; prior treatment-free interval (PTFI), defined as the time from completion of prior treatment to disease progression necessitating the current treatment; the current treatment line for PSRCC; regimen types and cycles of chemo or targeted therapy for the current treatment; and the duration of follow-up.

### Procedures

In the prospective cohort, LEER was performed according to the surgical procedures detailed in previous reports (7, 9). In brief, a midline incision was made on the abdomen, the bilateral paracolic gutters were incised, and the peritoneum was dissected at the base of the radix mesenterii for bowel mobilization. Then, the bilateral ureters were identified and liberated. If pelvic sidewall tumors had invaded the bladder and rectum, the bilateral paravesical and pararectal spaces and the space of Retzius were developed. The bilateral ureters were cut as close to the bladder as possible and the negative margins of the distal ureters were identified by frozen sections. Moreover, the mesosigmoid or mesorectum was skeletonized, and the blood vessels therein were ligated at a sufficient distance from the tumor. Bowel continuity was interrupted using a gastrointestinal anastomosis (GIA) staplers at the level of the proximal margin with no gross tumor.

For *en bloc* resection of pelvic sidewall tumors with negative resection margins, we first ligated the internal iliac artery just below the bifurcation of the common iliac artery and then divided the internal iliac vein at the bifurcation. The branches of the posterior division of the internal iliac vessel system, including the superior gluteal, inferior gluteal, and internal pudendal arteries and veins were transected using hemoclips or hemolock clips. Depending on the topography of PSRCC, the obturator internus muscle, the coccygeus muscle, and the levator ani muscles such as the pubococcygeus and iliococcygeus muscles were incised and separated from the pelvic sidewall with a Cobb periosteal dissector. Thereafter, the vulva was incised for removal of the urethra, lower vagina, and anus, and a dissection was carried to enter the space of Retizus and divide the pelvic floor musculature laterally and posteriorly. Recurrent pelvic sidewall tumors that were surrounded by the pelvic organs and adjacent pelvic floor muscles were removed through the inferior pelvic opening. After LEER, permanent colostomy and ileal conduit urinary diversion were carried out. In some cases, depending on the tumor location, the bladder, vagina and rectum could be preserved after checking the negative resection margin in frozen sections.

R0 resection was defined as lack of tumor invasion in the tissues of the lateral margins of the obturator internus, coccygeus, iliococcygeus and pubococcygeus muscles and the internal iliac vessel system ipsilateral to pelvic sidewall tumors on pathologic examination. If the bladder or rectum was preserved, an absence of tumor invasion in tissues surrounding the removed lesions according to multiple biopsies was considered R0 resection.

Postoperative complications were assessed by the Memorial Sloan Kettering Cancer Center criteria (16). For chemo or targeted therapy, single or combination regimens were used in both the prospective and retrospective cohorts. Moreover, targeted therapy using paclitaxel, cisplatin, and bevacizumab based on the Gynecologic Oncology Group (GOG) 240 trial was used beginning in August 2015 due to changes in insurance coverage (17, 18).

### Outcomes

The primary outcomes were the differences in treatment-free interval (TFI), progression-free survival (PFS), treatment-free survival (TFS), and overall survival (OS) between the prospective and retrospective cohorts. TFI was defined as the time interval from completion of treatment for PSRCC to disease progression; PFS was defined as the time interval from the start of treatment for PSRCC to disease progression; TFS was defined as the time interval from the start of treatment for PSRCC to cancer-related death or the end of the study; and OS was defined as the time interval from the diagnosis of cervical cancer to cancer-related death or the end of the study.

The secondary outcomes were the differences in tumor response, neurologic disturbance of the lower extremities, and severity of pelvic pain between the two groups. We assessed tumor response using the revised Response Evaluation Criteria in Solid Tumors (RECIST) version 1.1 (19), and neurologic disturbance of the lower extremities was evaluated by the severity of muscle weakness and neuralgia in the lower limbs according to the Common Terminology Criteria for Adverse Events (CTCAE) version 4.0. Moreover, pelvic pain severity was evaluated using both a numerical rating scale (NRS) and the number of morphine milligram equivalents (MME), representing the total amount of various opioids prescribed to control pelvic pain (20).

### Statistical Analysis

We compared non-parametric variables between the two groups with Mann-Whitney U, chi-square, and Fisher’s exact tests. Moreover, we compared TFI, PFS, TRS, and OS between the two groups by Kaplan-Meier survival analysis with the log-rank and Breslow tests and identified factors affecting survival using univariate and multivariate Cox proportional hazard regression models. All statistical tests were two-sided, and P < 0.05 was considered statistically significant. SPSS software version 21.0 (SPSS Inc., Chicago, IL, USA) was used.

## Results

### Study Population

**Figure 1:**
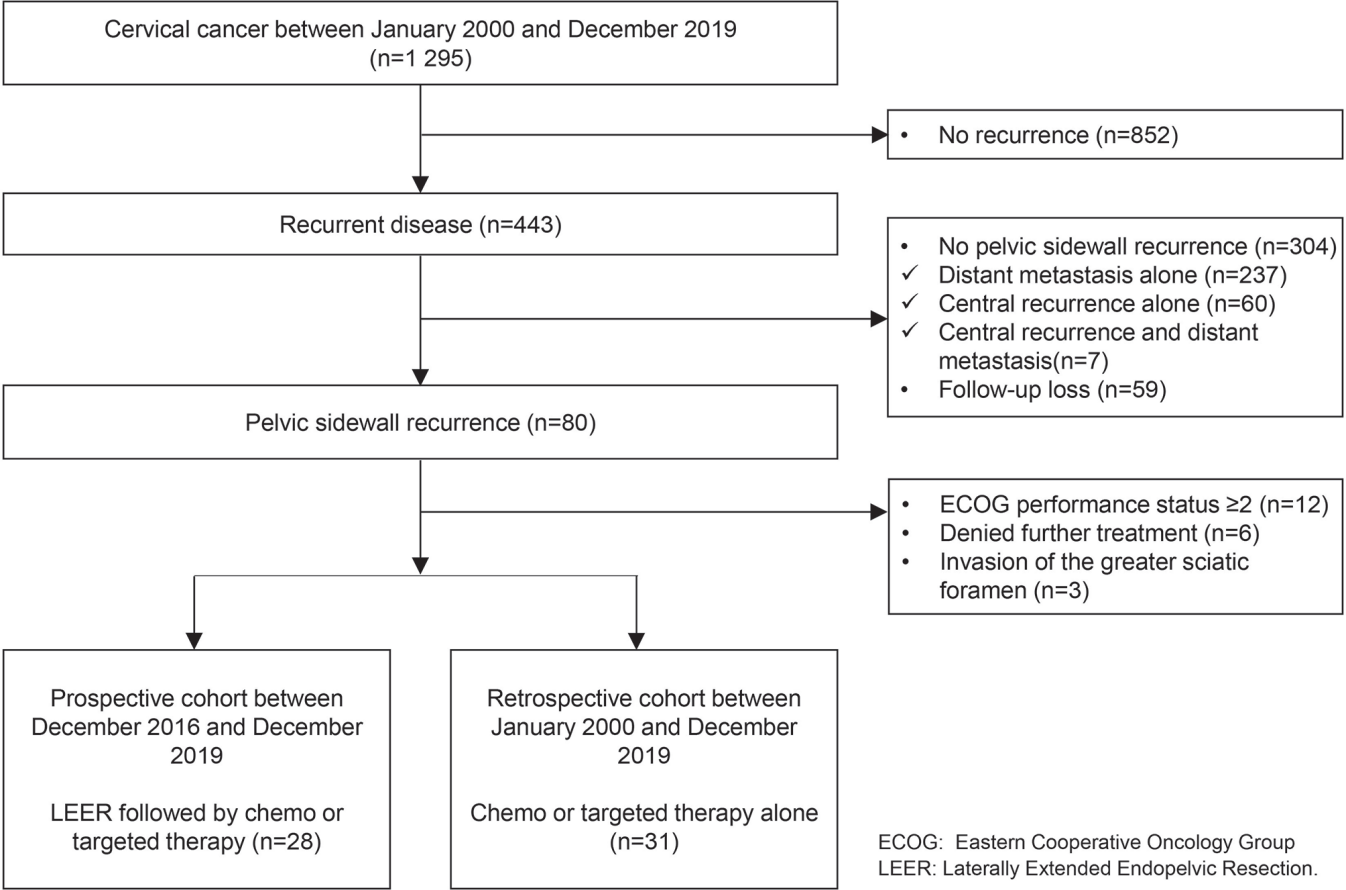
Diagram of the study flow.

Among 1,295 patients with cervical cancer, 443 (34.2%) showed disease recurrence. Of patients with disease recurrence, we excluded those with distant metastasis alone (n = 237, 18.3%), central recurrence alone (n = 60, 4.6%), and central recurrence and distant metastasis (n = 7, 0.5%); also, 59 (4.6%) were lost to follow-up. Among the remaining 80 patients with PSRCC (6.2%), we also excluded 12 (0.9%), six (0.5%), and three patients (0.2%) due to an ECOG performance status of two or more, denial of further treatment, and invasion of the greater sciatic foramen, respectively. Finally, we included 28 (2.2%) and 31 (2.4%) patients in the prospective and retrospective cohorts, respectively (**Figure 1**).

**Table 1** shows the clinico-pathologic characteristics of the study subjects. There were no differences in age, histologic types, size of pelvic sidewall tumors on imaging studies, radiologic TNM stage, topographic location and direction of pelvic sidewall tumors, types of prior treatment, tumor response to prior treatment, PTFI, or duration of follow-up between the two groups. In the prospective cohort, only 21 patients (75%) received LEER immediately after being diagnosed with PSRCC, whereas seven (25%) received second- or third-line chemo or targeted therapy prior to LEER. After LEER, three patients (10.7%) did not receive chemo or targeted therapy due to renal failure (n = 1) and rapid disease progression during management of postoperative complications (n = 2). Although there was no difference in the types of treatment regimens between the two groups, combination therapy using paclitaxel, cisplatin and bevacizumab was more common in the prospective cohort than in the retrospective cohort, and more cycles of chemo or targeted therapy were administered in the retrospective cohort than in the prospective cohort (**Table 2**).

**Table 1:** Clinicopathologic characteristics.

Characteristics	Prospective cohort (n=28)	Retrospective cohort (n=31)	P value
Age (years)	44.5 (28-70)	47 (31-71)	0.76
Histological types			0.47
Squamous cell carcinoma	21 (75)	26 (83.9)	
Endocervical adenocarcinoma	3 (10.7)	4 (12.9)	
Mucinous adenocarcinoma	2 (7.1)	0 (0)	
Adenosquamous carcinoma	1 (3.6)	1 (3.2)	
Large cell neuroendocrine tumor	1 (3.6)	0 (0)	
FIGO stage			0.829
Stage I	15 (53.6)	14 (45.2)	
Stage II	7 (25)	7 (22.6)	
Stage III	3 (10.7)	5 (16.1)	
Stage IV	3 (10.7)	5 (16.1)	
Size of pelvic sidewall tumor on imaging studies (cm)	3.5 (1.7-7.7)	3.6 (1-9.7)	0.83
Radiologic TNM stage			
T - Tumor			0.24
rT3b	23 (82.1)	29 (93.5)	
rT4	5 (17.9)	2 (6.5)	
N – Regional lymph nodes			0.46
rN0	18 (64.3)	17 (54.8)	
rN1	10 (35.7)	14 (45.2)	
M – Distant metastasis			0.54
rM0	21 (75)	21 (67.7)	
rM1	7 (25)	19 (32.3)	
Topographic location of pelvic sidewall tumor			0.27
Infra-iliac ischiopubic	2 (7.1)	0 (0)	
Infra-iliac acetabular	14 (50)	23 (74.2)	
Peri-iliac acetabular	2 (7.1)	2 (6.5)	
Infra-iliac sacrococcygeal	9 (32.1)	5 (16.1)	
Peri-iliac iliosacral	1 (3.6)	1 (3.2)	
Direction of pelvic sidewall tumor			0.42
Right	15 (53.6)	14 (45.2)	
Left	13 (46.4)	17 (54.8)	
Types of prior treatment			0.83
CCRT	3 (10.7)	0 (0)	
Surgery and chemoradiation	5 (17.9)	7 (22.6)	
Chemoradiation and chemotherapy	3 (10.7)	10 (32.3)	
Surgery, chemoradiation and chemotherapy	17 (60.7)	14 (45.2)	
Tumor response to prior treatment			0.89
Complete response	10 (47.6)	3 (42.9)	
Partial response	4 (19)	1 (14.3)	
Progressive disease	7 (33.3)	3 (42.9)	
Prior treatment-free interval (months)	9.3 (0.5, 321.5)	7.5 (0.6, 158.5)	0.89
Current treatment line for pelvic sidewall tumor			0.01
1	21 (75)	31 (100)	
2	5 (17.9)	0 (0)	
3	2 (7.1)	0 (0)	
Use of bevacizumab			0.02
No	14 (50)	25 (80.6)	
Before the current treatment	12 (42.9)	2 (6.5)	
During the current treatment	2 (7.1)	3 (9.7)	
After the current treatment	0 (0)	1 (3.2)	
Duration of follow-up (months)	36.7 (14.5-331.7)	35.7 (9.4-196.2)	0.51

Data are median (range) or n (%).

Patients in the prospective cohort received laterally extended endopelvic resection followed by chemo or targeted therapy, whereas those in the retrospective cohort received chemo or targeted therapy alone for pelvic sidewall recurrence of cervical cancer.

**Table 2:** Regimen type and number of cycles of chemo or targeted therapy for the current treatment.

	Prospective cohort (n = 28)	Retrospective cohort (n = 31)	P value
Types			0.13
No	3 (10.7)	0 (0)	
Paclitaxel/carboplatin	3 (10.7)	12 (38.7)	
Paclitaxel/cisplatin	2 (7.1)	0 (0)	
Topotecan/cisplatin	9 (32.1)	8 (25)	
5-fluorouracil/cisplatin	2 (7.1)	2 (6.5)	
5-fluorouracil/carboplatin	1 (3.6)	1 (3.2)	
Gemcitabine	4 (14.3)	1 (3.2)	
Cisplatin	1 (3.6)	2 (6.5)	
Topotecan	1 (3.6)	0 (0)	
Etoposide	0 (0)	1 (3.2)	
Irionotecan	0 (0)	1 (3.2)	
Paclitaxel/cisplatin/bevacizumab	2 (7.1)	3 (9.7)	
Cycles	3.5 (2 - 6)	5 (3 - 15)	<0.03

Data are median (range) or n (%).

Patients in the prospective cohort received laterally extended endopelvic resection followed by chemo or targeted therapy, whereas those in the retrospective cohort received chemo or targeted therapy alone for pelvic sidewall recurrence of cervical cancer.

### Treatment Outcomes

In terms of surgical extents, we were able to preserve the rectum alone and both the rectum and bladder in ten (35.7%) and three patients (10.7%), respectively. Among the pelvic sidewall structures, the obturator internus, pubococcygeus, iliococcygeus, and coccygeus muscles were resected in nine (32.1%), 12 (42.9%), 16 (57.1%), and 15 patients (53.6%), respectively, and the internal iliac vessel system was removed in 27 patients (96.4%; **Table 3**).

**Table 3:** Surgical extent.

	Prospective cohort (n = 28)
Preservation of the pelvic organs	
No	15 (53.6)
Rectum alone	10 (35.7)
Bladder and rectum alone	3 (10.7)
Extent of resection	
Bladder and urethra	25 (89.3)
Rectum and anus	15 (53.6)
Uterus	18 (64.3)
Vagina	20 (71.4)
Perineum	15 (53.6)
Obturator internus muscle	9 (32.1)
Pubococcygeus muscle	12 (42.9)
Iliococcygeus muscle	16 (57.1)
Coccygeus muscle	15 (53.6)
Internal iliac vessel system	27 (96.4)
Estimated blood loss (ml)	1800 (400 - 16800)
Transfusion	4 (0 - 39)
Operation time (minutes)	465 (190 - 760)
Hospitalization (days)	22 (8 - 86)

Data are median (range) or n (%).

Patients in the prospective cohort received laterally extended endopelvic resection followed by chemo or targeted therapy.

With regard to pathologic outcomes related to LEER, the median value of the size of pelvic sidewall tumors was 4.6 cm, and we achieved R0 resection in 26 patients (92.9%). Among the pelvic sidewall structures, tumor involvement in the obturator internus, pubococcygeus, iliococcygeus, and coccygeus muscles was seen in five (17.9%), four (14.3%), six (21.4%), and four (14.3%) patients, respectively, and 14 (50%) showed tumor involvement in the internal iliac vessel system (**Table 4**).

**Table 4:** Pathologic outcomes.

	Prospective cohort (n = 28)
Size of pelvic sidewall tumors (cm)	4.6 (1 - 11)
Resection margin	
R0	26 (92.9)
R1	2 (7.1)
Extent of tumor involvement	
Bladder	17 (60.7)
Urethra	3 (10.7)
Rectum	11 (39.3)
Anus	7 (25)
Uterus	1 (3.6)
Vagina	16 (57.1)
Perineum	0 (0)
Obturator internus muscle	5 (17.9)
Pubococcygeus muscle	4 (14.3)
Iliococcygeus muscle	6 (21.4)
Coccygeus muscle	4 (14.3)
Internal iliac vessel system	14 (50)

Data are median (range) or n (%).

Patients in the prospective cohort received laterally extended endopelvic resection followed by chemo or targeted therapy.

Postoperative complications developed in 17 patients (60.7%) after LEER. Arterial or venous thrombus was the most common complication (14.2%). Moreover, grade 3 or 4 complications according to the MSKCC surgical secondary events grading system were observed in 14 patients (50%; **Table 5**). In the retrospective cohort, the recto-vaginal fistula developed in 5 patients (16.1%); of these, four patients (12.9%) received bevacizumab. The association between bevacizumab usage and fistula development was not statistically significant (p = 0.088).

**Table 5:** Postoperative complications.

	Prospective cohort (n = 28)
ypes	
No	11 (39.3)
Arterial or venous thrombus	4 (14.2)
Leakage from the anastomotic site	3 (10.7)
Infected lymphocele	2 (7.1)
Inflammatory pelvic fluid collection	2 (7.1)
Acute pyelonephritis	1 (3.6)
Hydronephrosis	1 (3.6)
Ileus	1 (3.6)
Renal stone	1 (3.6)
Paralysis of low extremity	1 (3.6)
Wound dehiscence	1 (3.6)
Grade	
0	11 (39.3)
2	3 (10.7)
3	12 (42.9)
4	2 (7.1)

Data are median (range) or n (%).

Patients in the prospective cohort received laterally extended endopelvic resection followed by chemo or targeted therapy.

### Survival

**Figure 2:**
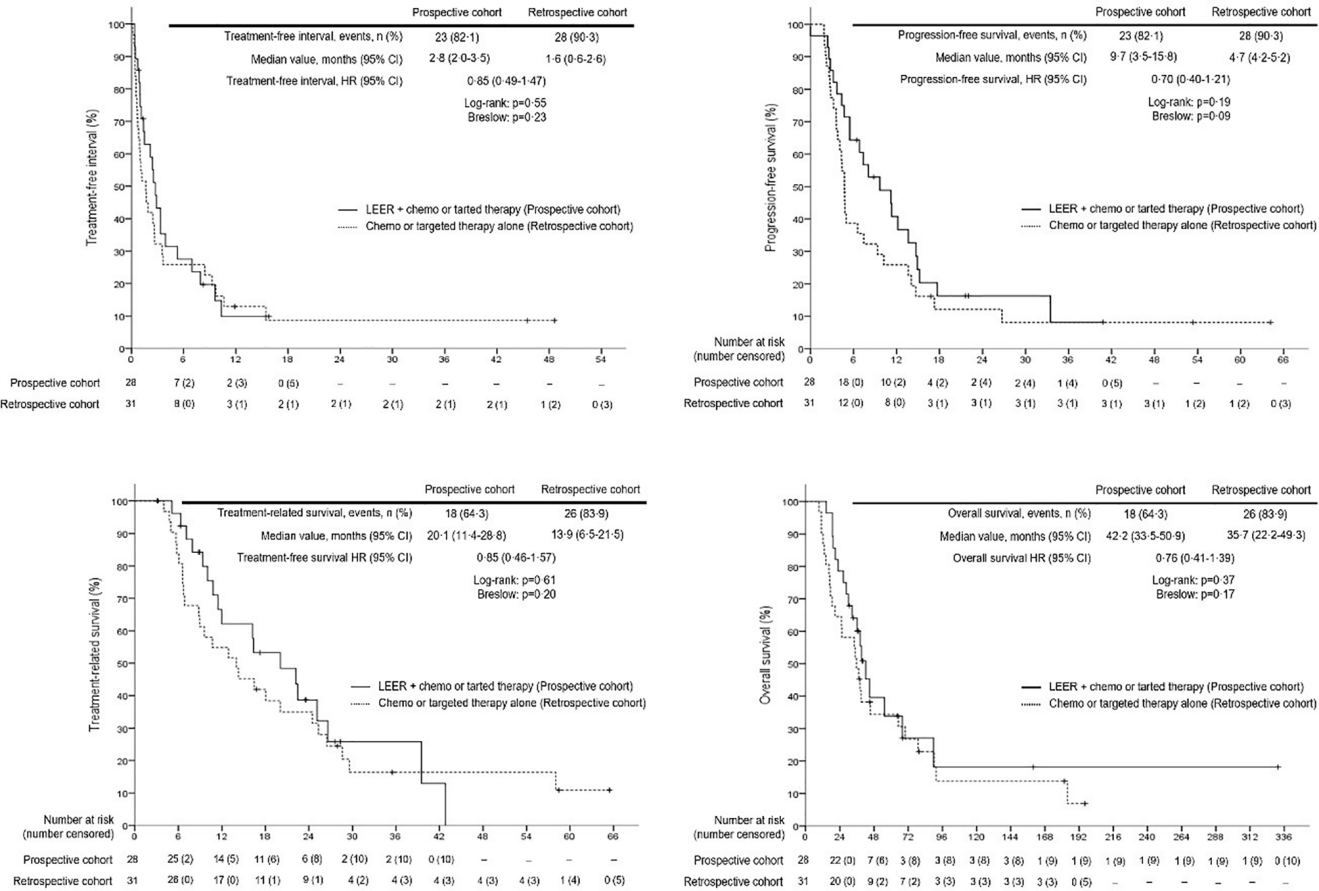
Comparison of treatment-free interval, progression-free survival, treatment-related survival and overall survival between the prospective and retrospective cohorts in all patients.

**Figure 3:**
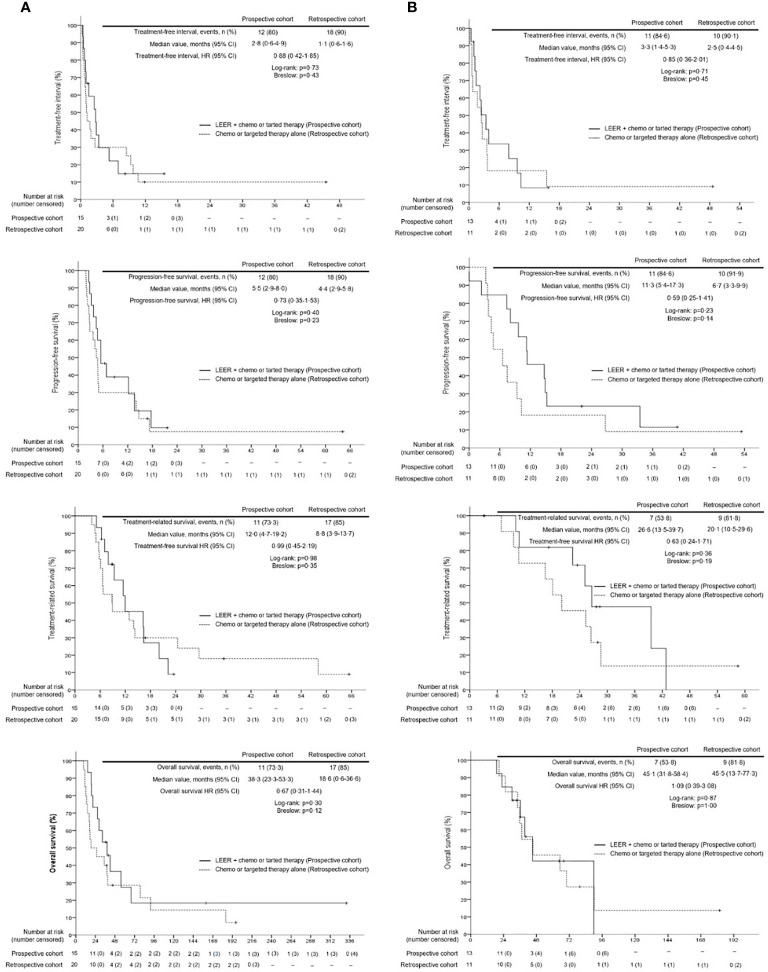
Comparison of treatment-free interval, progression-free survival, treatment-related survival and overall survival between the prospective and retrospective cohorts according to the favorable indication (tumor size ≤5 cm, prior treatment-free interval >5 months, and no distant metastasis) for laterally extended endopelvic resection; **(A)** unfavorable indication; **(B)** favorable indication.

**Figure 4:**
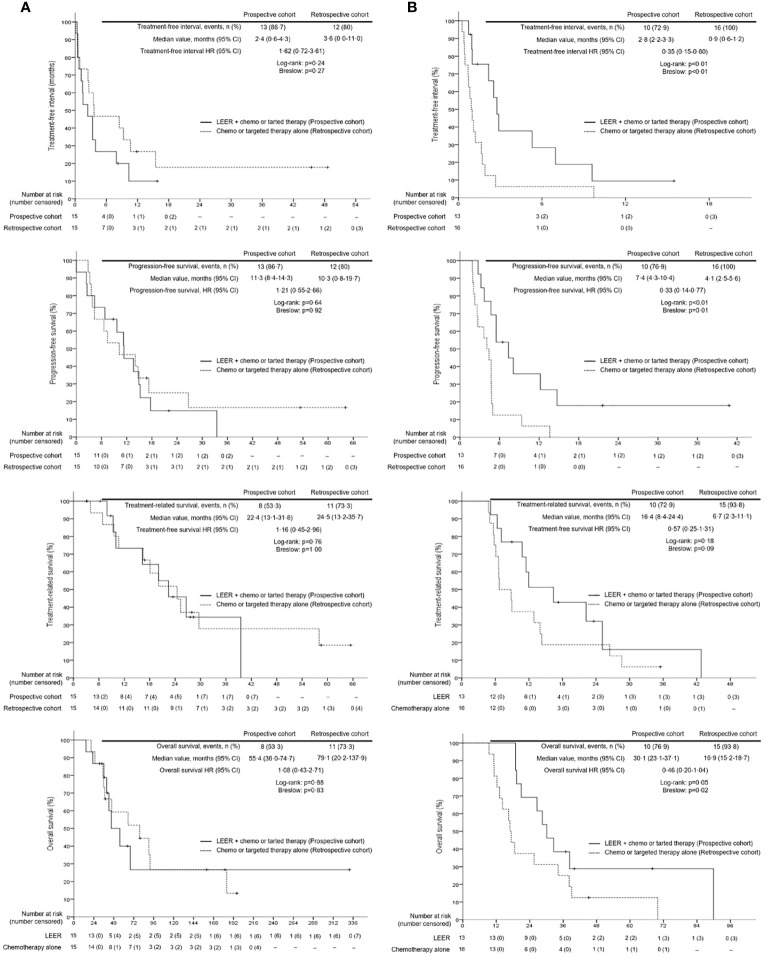
Comparison of treatment-free interval, progression-free survival, treatment-related survival and overall survival between patients treated with laterally extended endopelvic resection (LEER) followed by chemo or targeted therapy (prospective cohort) and those treated with chemo or targeted therapy alone (retrospective cohort) according to prior treatment-free interval: **(A)** ≥ 9.2 months and **(B)** < 9.2 months.

Survival analysis between the two groups revealed no differences in TFI, PFS, TRS, and OS between the prospective and retrospective cohorts in all patients (**Figure 2**). To go into greater detail, we also performed subgroup analyses based on the following favorable indications according to previous reports: tumor size ≤ 5 cm; PFTI > 5 months; and no distant metastasis (8, 21). As a result, we also found no difference in TFI, PFS, TRS, and OS between the prospective and retrospective cohorts based on the favorable indications (**Figure 3**). Furthermore, we conducted subgroup analyses based on the median value of PTFI, 9.2 months. In the 30 patients with PTFI ≥ 9.2 months, there were no differences in TFI, PFS, TRS and OS between the two groups, whereas LEER followed by chemo or targeted therapy was associated with improved TFI, PFS, and OS compared to chemo or targeted therapy alone (median values, 2.8 *vs.* 0.9 months; 7.4 *vs.* 4.1 months; 30.1 *vs.* 16.9 months; P ≤ 0.05) in the 29 patients with PTFI < 9.2 months (**Figure 4**).

Next, we conducted univariate and multivariate analyses to identify factors affecting survival (**Supplementary Tables 1–3**). The results showed that PTFI ≥ 9.2 months and LEER followed by chemo or targeted therapy were associated with improved TFI, PFS, TRS, and OS in all patients. Moreover, first-line treatment for PSRCC improved TFI and TRS, and rT3b was related to better TRS and OS. However, previous use of bevacizumab was related to worse TRS. In the subgroup analyses based on median PTFI, rT3b and current use of bevacizumab were factors associated with improved TFI, PFS, TSR, and OS in the 30 patients with PTFI ≥ 9.2 months. Furthermore, first-line treatment for PSRCC improved TRS and OS, and squamous cell carcinoma was associated with better OS. Although LEER was related to better TRS, previous use of bevacizumab was associated with reduced TRS. In the 29 patients with PTFI < 9.2 months, LEER was associated with improved TFI, PFS, TRS, and OS. Moreover, first-line treatment for PSRCC was associated with improved TFI and tumor size < 4.2 cm on imaging studies was related to better TRS and OS (**Table 6**).

**Table 6:** Factors affecting survival.

	All (n = 59)	PTFI ≥ 9.2 months (n = 30)	PTFI < 9.2 months (n = 29)
Treatment-free interval	–	–	–
rT3b	–	0.04 (0.01 - 0.57)	–
PTFI ≥ 9.2 months	0.42 (0.23 - 0.78)	–	–
First-line treatment for PSRCC	0.28 (0.09 - 0.80)	–	0.18 (0.03 - 0.98)
Use of bevacizumab			
Current	–	0.13 (0.02 - 0.65)	–
LEER followed by chemo or targeted therapy	0.54 (0.28 - 0.98)	–	0.28 (0.12 - 0.68)
Progression-free survival			
rT3b	–	0.18 (0.03 - 0.97)	–
PTFI ≥ 9.2 months	0.47 (0.26 - 0.85)	–	–
Use of bevacizumab			
Current	–	0.26 (0.06 - 0.82)	–
LEER followed by chemo or targeted therapy	0.60 (0.33 - 0.83)	–	0.27 (0.11 - 0.66)
Treatment-related survival			
Tumor size < 4.2 cm on imaging studies	–	–	0.41 (0.17 - 0.96)
rT3b	0.22 (0.08 - 0.57)	0.03 (0.02 - 0.58)	–
PTFI ≥ 9.2 months	0.51 (0.27 - 0.98)	–	–
First-line treatment for PSRCC	0.29 (0.10 - 0.88)	0.10 (0.01 - 0.76)	–
Use of bevacizumab			
Previous	3.28 (1.21 - 8.86)	5.48 (1.12 - 34.01)	–
Current	–	0.02 (0.01 - 0.36)	–
LEER followed by chemo or targeted therapy	0.25 (0.09 - 0.68)	0.15 (0.02 - 0.84)	0.44 (0.18 - 0.83)
Overall survival			
Squamous cell carcinoma	–	0.09 (0.01 - 0.58)	–
Tumor size < 4.2 cm on imaging studies	–	–	0.38 (0.16 - 0.89)
rT3b	0.24 (0.09 - 0.61)	0.23 (0.01 - 0.32)	–
PTFI ≥ 9.2 months	0.28 (0.14 - 0.55)	–	–
First-line treatment for PSRCC	–	0.06 (0.01 - 0.69)	–
Use of bevacizumab			–
Current	–	0.12 (0.02-0.79)	–
LEER followed by chemo or targeted therapy	0.50 (0.09 - 061)		0.37 (0.15 - 0.88)

Data are adjusted hazard ratio (95% confidence interval).

LEER, laterally extended endopelvic resection; PSRCC, pelvic sidewall recurrence of cervical cancer; PTFI, prior treatment-free interval.

### Tumor Response, Neurologic Disturbance and Pelvic Pain Severity

In terms of tumor response, complete response was more common in the prospective cohort than in the retrospective cohort (55.6 *vs.* 19.4%; P < 0.01). Despite the lack of differences in disease recurrence and death between the two groups, the prospective cohort showed a lower rate of PSRCC (25 *vs.* 67.7%; P = 0.01) and a higher rate of distant metastasis (53.6 *vs.* 6.5%; P = 0.01) than the retrospective cohort. Although the incidence of muscle weakness after treatment did not differ between the two groups, neuralgia was more common in the prospective cohort than in the retrospective cohort (50 *vs.* 12.9%; P < 0.01). However, there was no difference in grade 3 neuralgia between the two groups (3.6 *vs.* 0%; P = 0.48). Regarding pelvic pain severity, the lowest and highest NRS did not differ before and after treatment between the two groups. Although there was also no difference in the MME required to control pelvic pain before treatment between the two groups, the MME required to control pelvic pain after treatment was less in the prospective cohort than in the retrospective cohort (median, 0 *vs.* 15; P < 0.01; **Table 7**).

**Table 7:** Treatment outcomes.

Characteristics	Prospective cohort (n = 28)	Retrospective cohort (n = 31)	P value
Tumor response			<0.01
Complete response	15 (55.6)	6 (19.4)	
Partial response	0 (0)	4 (12.9)	
Progressive disease	12 (44.4)	21 (67.7)	
Disease recurrence	23 (82.1)	28 (90.3)	0.46
Recurrent sites			0.01
Central	1 (3.6)	4 (12.9)	
Pelvic sidewall	7 (25)	21 (67.7)	
Ipsilateral	6 (21.4)	21 (67.7)	
Contralateral	1 (3.6)	0 (0)	
Distant	15 (53.6)	2 (6.5)	
Death	18 (64.3)	26 (83.9)	0.08
Neurologic disturbance of low extremity			
Muscle weakness			0.06
No	22 (78.6)	31 (100)	
Grade 1	1 (3.6)	0 (0)	
Grade 2	2 (7.1)	0 (0)	
Grade 3	3 (10.7)	0 (0)	
Neuralgia			0.01
No	14 (50)	27 (87.1)	
Grade 1	8 (28.6)	1 (3.2)	
Grade 2	5 (17.9)	3 (9.7)	
Grade 3	1 (3.6)	0 (0)	
Pelvic pain severity			
Pre-treatment NRS			
Lowest	2 (0 - 4)	2 (0 - 5)	0.86
Highest	3 (0 - 9)	3 (0 - 10)	0.90
Post-treatment NRS			
Lowest	0 (0 - 4)	0 (0 - 5)	0.35
Highest	3 (0 - 6)	3 (0 - 9)	0.37
Pre-treatment MME/day	0 (0 - 312)	0 (0 - 210)	0.40
Post-treatment MME/day	0 (0 - 60)	15 (0 – 219)	<0.01

Data are median (range) or n (%).

Patients in the prospective cohort received laterally extended endopelvic resection followed by chemo or targeted therapy, while those in the retrospective cohort received chemo or targeted therapy alone for pelvic sidewall recurrence of cervical cancer.

MME, morphine milligrams equivalents; NRS, numeral rating scale.

## Discussion

LEER has long been used to remove pelvic tumors within ontogenetic cancer fields and sustain loco-regional tumor control (22–24). LEER is done by resecting the pelvic floor and sidewall muscles and the internal iliac vessel system surrounding pelvic sidewall tumors. R0 resection is more common in patients treated with LEER than in those who undergo pelvic exenteration. Given these treatment options, the rates of five-year PFS and OS have been reported to reach 65% and 75%, respectively, in patients with relapsed pelvic malignancies (25). A recent multicenter study showed that achieving R0 resection during laterally extended pelvic resection is the most important prognostic factor for gynecologic malignancies involving pelvic sidewall (22). Previous research excluded recurrent gynecologic cancer patients who achieved a disease-free interval of less than 6 months, but there are no relevant published studies to evaluate the favorable indications for LEER (23, 26). Therefore, there is no evidence by which to judge the efficacy and safety of LEER compared to chemo or targeted therapy alone, which is a major limitation in generalizing the application of LEER for patients with PSRCC.

Although a previous study showed that the five-year OS and PFS rates were 46% and 35%, respectively, in patients with PSRCC without a therapeutic alternative to LEER (8), the current study demonstrated that the prognosis for such patients is relatively poor, with a two-year PFS rate of 16.3% and a similar five-year OS rate of 33.9%. This poor PFS after LEER is most likely because ten patients (35.7%) with regional lymph node metastasis and seven (25%) with distant metastasis were included in the prospective cohort. By contrast, in the previous study, only 22.2% of patients had regional lymph node metastasis without distant metastasis.

Furthermore, the favorable indications for LEER (tumor size ≤5 cm; PFTI >5 months; no distant metastasis) were not related to improved survival, and the prognosis of patients with these indications remained relatively poor, with a two-year PFS rate of 23.1% despite a similar OS rate of 42.1%. This poor prognosis may be related to the high potential for distant metastasis seen in PSRCC. Although pelvic sidewall tumors can infiltrate the remaining lymphatic vessels connected to lymph node basins in the pelvic viscero-parietal compartments (27, 28), complete resection of these compartments outside the scope of LEER is difficult because of severe fibrosis or adhesion due to previous surgery or radiotherapy, which can cause distant metastasis if tumor cells are present in these compartments.

The above explanation is supported by the finding that PSRCC was associated with a similar prognosis as distant metastasis in a previous study (4), and distant metastasis was found in 25% of patients with PSRCC in this study. On the other hand, if LEER was improperly implemented in this study, its use may be related to poor prognosis. However, the finding that 53.6% of patients with relapse after LEER showed distant metastasis supports the surgical suitability of LEER with appropriate loco-regional control.

The most important finding of this study is that LEER may be beneficial for the treatment of PSRCC in patients with PTFI <9.2 months. Patients with PTFI ≥9.2 months may have platinum sensitivity (29–31), which can increase tumor response to chemotherapy such that it matches the surgical effect of LEER. Since this study showed that targeted therapy using bevacizumab increased survival, as in the GOG 240 trial (18), combined chemotherapy with bevacizumab can be considered as a first-line treatment for PSRCC because its use avoids surgical complications and its efficacy is similar to that of LEER in patients with PTFI ≥ 9.2 months.

Importantly, LEER may be effective at reducing the MME required to control pelvic pain. Although opioid-based analgesic treatment can relieve pelvic pain in more than 70% of patients, many patients still suffer due to underutilization of opioids and the adverse effects of opioids (32). Since sciatica occurs when one or more nerve roots from L4 to S3 are compressed by pelvic sidewall tumors, tumor removal through LEER can relieve the pressure on nerve roots and markedly reduce the associated pain (33), which means that LEER can be considered as a palliative surgery for relief of uncontrolled sciatica caused by PSRCC (12).

This study has some limitations. First, the small number of enrolled patients and the heterogeneity of both cohorts due to the rarity of PSRCC may have introduced bias. Second, little relevant data was available with which to design this study and to calculate the appropriate sample size. Third, only the combination therapy using paclitaxel, cisplatin, and bevacizumab has been approved for recurrent cervical cancer since March 2015, whereas the use of bevacizumab monotherapy is not currently approved in our country. Thus, the rate of bevacizumab usage was low this study. Fourth, the comparison of pain severity should be interpreted carefully, considering the two different study designs. Fifth, we did not include bilateral pelvic sidewall recurrence, because bilateral LEER is insufficiently safe. Thus, it is essential to more clearly evaluate the efficacy and safety of LEER compared to chemo or targeted therapy alone through a multicenter study based on our results.

To the best of our knowledge, this is the first comparative study between LEER and chemo or targeted therapy alone for PSRCC. Compared to chemo or targeted therapy alone, LEER may improve survival, with increased tumor response in patients with PSRCC and PTFI < 9.2 months. Moreover, LEER may be an effective means of controlling the pelvic pain caused by PSRCC.

## Data Availability Statement

The raw data supporting the conclusions of this article will be made available by the authors, without undue reservation.

## Ethics Statement

The studies involving human participants were reviewed and approved by Seoul National University Hospital. The patients/participants provided their written informed consent to participate in this study. Written informed consent was obtained from the individual(s) for the publication of any potentially identifiable images or data included in this article.

## Author Contributions

SP: methodology, validation, formal analysis, investigation, data curation, writing-original draft, and visualization. JM: validation, investigation, and writing-review & editing. SL: validation, investigation, and writing-review & editing. YL: validation, investigation, and writing-review & editing. HC: methodology, validation, investigation, and supervision. J-WK: methodology, investigation, and writing-review & editing. NP: methodology, validation, investigation, and supervision. YS: methodology, validation, investigation, and supervision. HK: conceptualization, methodology, formal analysis, resources, writing - review & editing, supervision, project administration, and funding acquisition. All authors contributed to the article and approved the submitted version.

## Funding

This research was supported by grants from Seoul National University (No. 800-20200458; 800-20200309; 800-20190437).

## Conflict of Interest

The authors declare that the research was conducted in the absence of any commercial or financial relationships that could be construed as a potential conflict of interest.
