# Supplementary material for: Laterally Extended Endopelvic Resection Versus Chemo or Targeted Therapy Alone for Pelvic Sidewall Recurrence of Cervical Cancer
Source: Front Oncol. 2021 May 25;11:683441. doi: 10.3389/fonc.2021.683441 (PMC8186785; doi:10.3389/fonc.2021.683441)
Supplement: Supplementary file 1 [file Table_1.docx]

Supplementary Material

Supplementary Table 1. Factors affecting survival in all patients

| **Survival** | **Univariate** | | | **Multivariate** | | |
| --- | --- | --- | --- | --- | --- | --- |
|  | **HR** | **95% CI** | **P value** | **Adjusted HR** | **95% CI** | **P value** |
| *Treatment-free interval* |  |  |  |  |  |  |
| Age ≥48 years | 1.35 | 0.78 - 2.37 | 0.29 | .. | .. | .. |
| Squamous cell carcinoma | 1.13 | 0.58 - 2.20 | 0.73 | .. | .. | .. |
| Tumor size <4.2 cm on imaging studies | 1.10 | 0.63 - 1.93 | 0.74 | .. | .. | .. |
| rT3b | 0.53 | 0.23 - 1.19 | 0.12 | .. | .. | .. |
| rN0 | 0.85 | 0.49 - 1.49 | 0.58 | .. | .. | .. |
| rM0 | 1.20 | 0.65 - 2.23 | 0.56 | .. | .. | .. |
| PTFI ≥9.2 months | 0.56 | 0.32 - 0.98 | 0.04 | 0.42 | 0.23 - 0.78 | < 0.01 |
| First-line treatment for PSRCC | 0.47 | 0.18 - 1.22 | 0.12 | 0.28 | 0.09 - 0.80 | 0.02 |
| Use of bevacizumab |  |  |  |  |  |  |
| Previous | 1.41 | 0.72 - 2.75 | 0.32 | .. | .. | .. |
| Current | 0.55 | 0.20 - 1.56 | 0.26 | .. | .. | .. |
| LEER followed by chemo or targeted therapy | 0.85 | 0.49 - 1.47 | 0.55 | 0.54 | 0.28 - 0.98 | 0.04 |
| *Progression-free survival* |  |  |  |  |  |  |
| Age ≥48 years | 1.22 | 0.67 - 2.21 | 0.52 | .. | .. | .. |
| Squamous cell carcinoma | 1.50 | 0.72 - 3.14 | 0.28 | .. | .. | .. |
| Tumor size <4.2 cm on imaging studies | 1.20 | 0.65 - 2.22 | 0.56 | .. | .. | .. |
| rT3b | 0.52 | 0.20 - 1.35 | 0.18 | .. | .. | .. |
| rN0 | 0.81 | 0.44 - 1.49 | 0.50 | .. | .. | .. |
| rM0 | 1.25 | 0.64 - 2.43 | 0.52 | .. | .. | .. |
| PTFI ≥9.2 months | 0.54 | 0.29 - 0.99 | 0.04 | 0.47 | 0.26 - 0.85 | 0.01 |
| First-line treatment for PSRCC | 0.88 | 0.34 - 2.24 | 0.78 | .. | .. | .. |
| Use of bevacizumab |  |  |  |  |  |  |
| Previous | 0.99 | 0.47 - 2.10 | 0.99 | .. | .. | .. |
| Current | 0.67 | 0.23 - 1.90 | 0.67 | .. | .. | .. |
| LEER followed by chemo or targeted therapy | 0.67 | 0.36 - 1.25 | 0.21 | 0.60 | 0.33 - 0.83 | 0.04 |
| *Treatment-related survival* |  |  |  |  |  |  |
| Age ≥48 years | 1.23 | 0.68 - 2.24 | 0.49 | .. | .. | .. |
| Squamous cell carcinoma | 1.71 | 0.79 - 3.69 | 0.17 | .. | .. | .. |
| Tumor size <4.2 cm on imaging studies | 0.68 | 0.37 - 1.24 | 0.21 | .. | .. | .. |
| rT3b | 0.29 | 0.12 - 0.68 | < 0.01 | 0.22 | 0.08 - 0.57 | < 0.01 |
| rN0 | 0.68 | 0.37 - 1.24 | 0.21 | .. | .. | .. |
| rM0 | 1.11 | 0.58 - 2.11 | 0.76 | .. | .. | .. |
| PTFI ≥9.2 months | 0.44 | 0.24 - 0.82 | <0.01 | 0.51 | 0.27 - 0.98 | 0.04 |
| First-line treatment for PSRCC | 0.50 | 0.19 - 1.29 | 0.15 | 0.29 | 0.10 - 0.88 | 0.03 |
| Use of bevacizumab |  |  |  |  |  |  |
| Previous | 1.33 | 0.67 - 2.63 | 0.42 | 3.28 | 1.21-8.86 | 0.02 |
| Current | 0.46 | 0.14 - 1.50 | 0.20 | .. | .. | .. |
| LEER followed by chemo or targeted therapy | 0.85 | 0.46 - 1.57 | 0.61 | 0.25 | 0.09 - 0.68 | < 0.01 |
| *Overall survival* |  |  |  |  |  |  |
| Age ≥48 years | 2.12 | 1.11 - 4.07 | 0.02 | .. | .. | .. |
| Squamous cell carcinoma | 1.36 | 0.63 - 2.95 | 0.44 | .. | .. | .. |
| Tumor size <4.2 cm on imaging studies | 0.80 | 0.44 - 1.45 | 0.46 | .. | .. | .. |
| rT3b | 0.27 | 0.12 - 0.63 | < 0.01 | 0.24 | 0.09 - 0.61 | < 0.01 |
| rN0 | 0.94 | 0.52 - 1.71 | 0.84 | .. | .. | .. |
| rM0 | 1.05 | 0.55 - 1.99 | 0.88 | .. | .. | .. |
| PTFI ≥9.2 months | 0.29 | 0.15 - 0.55 | < 0.01 | 0.28 | 0.14 - 0.55 | < 0.01 |
| First-line treatment for PSRCC | 0.69 | 0.27 – 1.76 | 0.44 | .. | .. | .. |
| Use of bevacizumab | 1.40 | 0.69 - 2.82 | 0.35 |  |  |  |
| Previous | 1.34 | 0.70 - 2.82 | 0.35 | .. | .. | .. |
| Current | 0.78 | 0.24 - 2.60 | 0.69 | .. | .. | .. |
| LEER followed by chemo or targeted therapy | 0.76 | 0.41 - 1.39 | 0.37 | 0.50 | 0.09 - 0.61 | 0.04 |

Abbreviation: CI, confidence interval; HR, hazard ratio; LEER, laterally extended endopelvic resection; PSRCC, pelvic sidewall recurrence of cervical cancer; PTFI, prior treatment-free interval.

Supplementary Table 2. Factors affecting survival in 30 patients with PTFI ≥9.2 months

| Survival | Univariate | | | Multivariate | | |
| --- | --- | --- | --- | --- | --- | --- |
|  | HR | 95% CI | P value | Adjusted HR | 95% CI | P value |
| *Treatment-free interval* |  |  |  |  |  |  |
| Age ≥48 years | 1.87 | 0.82 - 4.25 | 0.14 | .. | .. | .. |
| Squamous cell carcinoma | 0.92 | 0.38 - 2.21 | 0.85 | .. | .. | .. |
| Tumor size <4.2 cm on imaging studies | 1.63 | 0.69 - 3.79 | 0.26 | .. | .. | .. |
| rT3b | 0.06 | 0.01 - 0.46 | <0.01 | 0.04 | 0.01 - 0.57 | 0.02 |
| rN0 | 0.87 | 0.39 - 1.95 | 0.74 | .. | .. | .. |
| rM0 | 0.58 | 0.23 - 1.48 | 0.26 | .. | .. | .. |
| First-line treatment after the pelvic sidewall recurrence | 0.33 | 0.09 - 1.20 | 0.09 | .. | .. | .. |
| Use of bevacizumab |  |  |  |  |  |  |
| Previous | 4.05 | 1.36 - 12.09 | 0.01 | .. | .. | .. |
| Current | 0.36 | 0.08 - 1.56 | 0.17 | 0.13 | 0.02 - 0.65 | 0.01 |
| LEER followed by chemo or targeted therapy | 1.62 | 0.72 - 3.61 | 0.24 | .. | .. | .. |
| *Progression-free survival* |  |  |  |  |  |  |
| Age ≥48 years | 1.03 | 0.46 - 2.32 | 0.94 | .. | .. | .. |
| Squamous cell carcinoma | 1.11 | 0.46 - 2.71 | 0.82 | .. | .. | .. |
| Tumor size <4.2 cm on imaging studies | 1.75 | 0.75 - 4.09 | 0.20 | .. | .. | .. |
| rT3b | 0.24 | 0.05 - 1.10 | 0.07 | 0.18 | 0.03 - 0.97 | 0.04 |
| rN0 | 0.91 | 0.41 - 2.01 | 0.81 | .. | .. | .. |
| rM0 | 1.95 | 0.77 - 4.94 | 0.16 | .. | .. | .. |
| First-line treatment after the pelvic sidewall recurrence | 0.60 | 0.17 - 2.11 | 0.42 | .. | .. | .. |
| Use of bevacizumab |  |  |  |  |  |  |
| Previous | 0.92 | 0.33 - 2.52 | 0.87 | .. | .. | .. |
| Current | 0.40 | 0.09 - 1.76 | 0.23 | 0.26 | 0.06 - 0.82 | 0.04 |
| LEER followed by chemo or targeted therapy | 1.21 | 0.55 - 2.66 | 0.64 | .. | .. | .. |
| *Treatment-related survival* |  |  |  |  |  |  |
| Age ≥48 years | 1.13 | 0.46 - 2.82 | 0.79 | .. | .. | .. |
| Squamous cell carcinoma | 1.36 | 0.49 - 3.81 | 0.55 | .. | .. | .. |
| Tumor size <4.2 cm on imaging studies | 0.94 | 0.36 - 2.46 | 0.90 | .. | .. | .. |
| rT3b | 0.07 | 0.01 - 0.51 | <0.01 | 0.03 | 0.02 - 0.58 | 0.02 |
| rN0 | 1.56 | 0.63 - 3.88 | 0.34 | .. | .. | .. |
| rM0 | 1.27 | 0.47 - 3.42 | 0.64 | .. | .. | .. |
| First-line treatment after the pelvic sidewall recurrence | 0.18 | 0.04 - 0.75 | 0.02 | 0.10 | 0.01 - 0.76 | 0.03 |
| Use of bevacizumab |  |  |  |  |  |  |
| Previous | 2.01 | 0.70 - 8.75 | 0.20 | 5.48 | 1.12 - 34.01 | 0.04 |
| Current | 0.30 | 0.04 - 2.29 | 0.24 | 0.02 | 0.01 - 0.36 | < 0.01 |
| LEER followed by chemo or targeted therapy | 1.16 | 0.45 - 2.96 | 0.76 | 0.15 | 0.02 - 0.84 | 0.03 |
| *Overall survival* |  |  |  |  |  |  |
| Age ≥48 years | 2.44 | 0.90 - 6.65 | 0.08 | .. | .. | .. |
| Squamous cell carcinoma | 0.71 | 0.25 - 2.03 | 0.53 | 0.09 | 0.01 - 0.58 | 0.01 |
| Tumor size <4.2 cm on imaging studies | 1.53 | 0.60 -3.95 | 0.37 | .. | .. | .. |
| rT3b | 0.20 | 0.04 - 0.93 | 0.04 | 0.23 | 0.01 - 0.32 | < 0.01 |
| rN0 | 0.90 | 0.36 - 2.23 | 0.82 | .. | .. | .. |
| rM0 | 1.36 | 0.51 - 3.67 | 0.54 | .. | .. | .. |
| First-line treatment after the pelvic sidewall recurrence | 0.27 | 0.07 -0.97 | 0.04 | 0.06 | 0.01 - 0.69 | 0.02 |
| Use of bevacizumab |  |  |  |  |  |  |
| Previous | 2.38 | 0.79 - 7.15 | 0.12 | .. | .. | .. |
| Current | 0.60 | 0.08 - 4.69 | 0.63 | 0.12 | 0.02 - 0.79 | 0.03 |
| LEER followed by chemo or targeted therapy | 1.08 | 0.43 - 2.71 | 0.88 | .. | .. | .. |

Abbreviation: CI, confidence interval; HR, hazard ratio; LEER, laterally extended endopelvic resection; PTFI, prior treatment-free interval.

Supplementary Table 3. Factors affecting survival in 29 patients with PTFI <9.2 months

| Survival | Univariate | | | Multivariate | | |
| --- | --- | --- | --- | --- | --- | --- |
|  | HR | 95% CI | P value | Adjusted HR | 95% CI | P value |
| *Treatment-free interval* |  |  |  |  |  |  |
| Age ≥48 years | 0.61 | 0.27 - 1.37 | 0.23 | .. | .. | .. |
| Squamous cell carcinoma | 1.32 | 0.45 - 3.87 | 0.62 | .. | .. | .. |
| Tumor size <4.2 cm on imaging studies | 0.75 | 0.33 - 1.69 | 0.48 | .. | .. | .. |
| rT3b | 1.10 | 0.41 - 2.95 | 0.85 | .. | .. | .. |
| rN0 | 0.76 | 0.33 - 1.76 | 0.52 | .. | .. | .. |
| rM0 | 1.30 | 0.55 - 3.08 | 0.55 | .. | .. | .. |
| First-line treatment after the pelvic sidewall recurrence | 0.43 | 0.09 - 1.95 | 0.27 | 0.18 | 0.03 - 0.98 | 0.04 |
| Use of bevacizumab |  |  |  |  |  |  |
| Previous | 0.65 | 0.26 - 1.57 | 0.35 | .. | .. | .. |
| Current | 7.07 | 1.33 - 37.49 | 0.02 | .. | .. | .. |
| LEER followed by chemo or targeted therapy | 0.35 | 0.15 - 0.80 | 0.01 | 0.28 | 0.12 - 0.68 | < 0.01 |
| *Progression-free survival* |  |  |  |  |  |  |
| Age ≥48 years | 0.78 | 0.35 - 1.73 | 0.54 | .. | .. | .. |
| Squamous cell carcinoma | 1.52 | 0.52 - 4.47 | 0.44 | .. | .. | .. |
| Tumor size <4.2 cm on imaging studies | 0.61 | 0.28 - 1.33 | 0.22 | .. | .. | .. |
| rT3b | 0.84 | 0.31 - 2.24 | 0.72 | .. | .. | .. |
| rN0 | 0.58 | 0.25 - 1.35 | 0.21 | .. | .. | .. |
| rM0 | 0.55 | 0.22 - 1.34 | 0.19 | .. | .. | .. |
| First-line treatment after the pelvic sidewall recurrence | 0.98 | 0.23 - 4.22 | 0.98 | .. | .. | .. |
| Use of bevacizumab |  |  |  |  |  |  |
| Previous | 0.69 | 0.28 - 1.69 | 0.42 | .. | .. | .. |
| Current | 2.30 | 0.51 - 10.42 | 0.28 | .. | .. | .. |
| LEER followed by chemo or targeted therapy | 0.33 | 0.14 - 0.77 | 0.01 | 0.27 | 0.11 - 0.66 | < 0.01 |
| *Treatment-related survival* |  |  |  |  |  |  |
| Age ≥48 years | 0.89 | 0.38 - 2.01 | 0.78 | .. | .. | .. |
| Squamous cell carcinoma | 1.86 | 0.55 - 6.28 | 0.32 | .. | .. | .. |
| Tumor size <4.2 cm on imaging studies | 0.52 | 0.23 - 1.16 | 0.11 | 0.41 | 0.17 - 0.96 | 0.04 |
| rT3b | 0.46 | 0.17 - 1.25 | 0.13 | .. | .. | .. |
| rN0 | 1.65 | 0.71 - 3.87 | 0.25 | .. | .. | .. |
| rM0 | 0.50 | 0.20 - 1.25 | 0.14 | .. | .. | .. |
| First-line treatment after the pelvic sidewall recurrence | 0.80 | 0.18 -3.47 | 0.76 | .. | .. | .. |
| Use of bevacizumab |  |  |  |  |  |  |
| Previous | 0.71 | 0.28 - 1.83 | 0.48 | .. | .. | .. |
| Current | 0.63 | 0.14 - 2.78 | 0.54 | .. | .. | .. |
| LEER followed by chemo or targeted therapy | 0.57 | 0.25 - 1.31 | 0.19 | 0.44 | 0.18 - 0.83 | 0.04 |
| *Overall survival* |  |  |  |  |  |  |
| Age ≥48 years | 1.06 | 0.45 - 2.50 | 0.89 | .. | .. | .. |
| Squamous cell carcinoma | 2.28 | 0.66 - 7.87 | 0.19 | .. | .. | .. |
| Tumor size <4.2 cm on imaging studies | 0.41 | 0.18 - 0.96 | 0.03 | 0.38 | 0.16 - 0.89 | 0.03 |
| rT3b | 0.46 | 0.16 - 1.29 | 0.14 | .. | .. | .. |
| rN0 | 0.93 | 0.41 - 2.14 | 0.87 | .. | .. | .. |
| rM0 | 0.65 | 0.28 - 1.56 | 0.34 | .. | .. | .. |
| First-line treatment after the pelvic sidewall recurrence | 3.30 | 0.43 - 25.53 | 0.25 | .. | .. | .. |
| Use of bevacizumab |  |  |  |  |  |  |
| Previous | 0.74 | 0.30 - 1.84 | 0.52 | .. | .. | .. |
| Current | 1.28 | 0.29 - 5.70 | 0.75 | .. | .. | .. |
| LEER followed by chemo or targeted therapy | 0.45 | 0.20 - 1.04 | 0.06 | 0.37 | 0.15 - 0.88 | 0.02 |

Abbreviation: CI, confidence interval; HR, hazard ratio; LEER, laterally extended endopelvic resection; PTFI.
